# Supplementary figures and images for: Episodic future thinking in type 2 diabetes: Further development and validation of the Health Information Thinking control for clinical trials
Source: PLoS One. 2023 Aug 3;18(8):e0289478. doi: 10.1371/journal.pone.0289478 (PMC10399790; doi:10.1371/journal.pone.0289478)

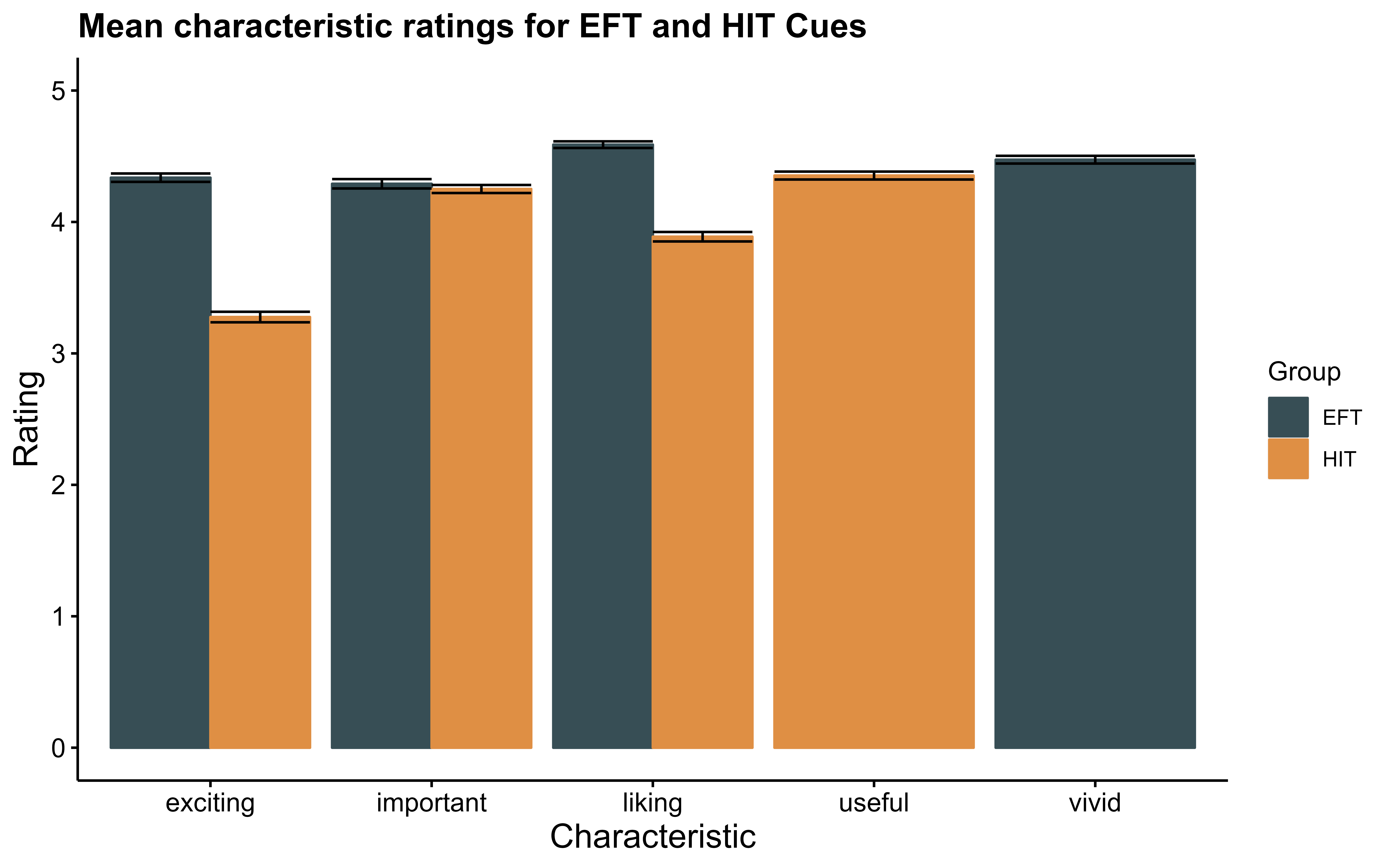

Supplement: S3 Fig — (PNG) [file pone.0289478.s003.png]
