# Supplementary material for: Episodic future thinking in type 2 diabetes: Further development and validation of the Health Information Thinking control for clinical trials
Source: PLoS One. 2023 Aug 3;18(8):e0289478. doi: 10.1371/journal.pone.0289478 (PMC10399790; doi:10.1371/journal.pone.0289478)
Supplement: S1 Table — (PDF) [file pone.0289478.s004.pdf]

Variable	Overall, N = 524[1]	Completion Status		p-value[2]
		**Completed**, N = 434[1]	**Voluntarily Withdrawn**, N = 90[1]	
**Group**				<0.001
EFT	174 / 524 (33%)	120 / 434 (28%)	54 / 90 (60%)	
HIT	175 / 524 (33%)	142 / 434 (33%)	33 / 90 (37%)	
NCC	175 / 524 (33%)	172 / 434 (40%)	3 / 90 (3.3%)	
**Age**	44.00 (35.00, 53.00)	44.00 (35.00, 53.75)	41.50 (35.00, 49.75)	0.2
**BMI**	37.26 (33.45, 43.75)	37.12 (33.40, 43.31)	37.61 (33.97, 44.66)	0.5
**Income**	34,999.50 (4,999.50, 54,999.50)	34,999.50 (4,999.50, 54,999.50)	34,999.50 (9,999.50, 54,999.50)	0.4
Unknown	15	11	4	
**Contemplation Ladder**	9.00 (8.00, 10.00)	9.00 (8.00, 10.00)	9.00 (8.00, 10.00)	0.9
Unknown	5	4	1	
**HbA1c**				0.3
6.9% or lower	210 / 524 (40%)	168 / 434 (39%)	42 / 90 (47%)	
7.0% - 8.0%	151 / 524 (29%)	125 / 434 (29%)	26 / 90 (29%)	
8.0% - 8.9%	56 / 524 (11%)	46 / 434 (11%)	10 / 90 (11%)	
9.0% or greater	41 / 524 (7.8%)	38 / 434 (8.8%)	3 / 90 (3.3%)	
Unkown by participant	66 / 524 (13%)	57 / 434 (13%)	9 / 90 (10%)	
**Gender**				0.4
Female	319 / 524 (61%)	269 / 434 (62%)	50 / 90 (56%)	
Male	202 / 524 (39%)	162 / 434 (37%)	40 / 90 (44%)	

[1] n / N (%); Median (IQR)

[2] Pearson's Chi-squared test; Wilcoxon rank sum test; Fisher's exact test

**Variable**	**Overall**, N = 524[1]	**Completion Status** **Completed**, N = 434[1]	**Voluntarily Withdrawn**, N = 90[1]	**p-value**[2]
Other (please specify)	3 / 524 (0.6%)	3 / 434 (0.7%)	0 / 90 (0%)	

[1] n / N (%); Median (IQR)

[2] Pearson's Chi-squared test; Wilcoxon rank sum test; Fisher's exact test
