# Supplementary material for: Episodic future thinking in type 2 diabetes: Further development and validation of the Health Information Thinking control for clinical trials
Source: PLoS One. 2023 Aug 3;18(8):e0289478. doi: 10.1371/journal.pone.0289478 (PMC10399790; doi:10.1371/journal.pone.0289478)
Supplement: S1 Appendix — (PDF) [file pone.0289478.s005.pdf]

Supplementary Materials

Sensitivity analysis of results: Ordinal AUC as a function of group assignment.

Results of the one-way ANOVA examining differences in ordinal AUC between groups (including all participants who completed the experiment; n = 120, n = 142, and n = 172 for EFT, HIT, and NCC groups, respectively) indicate that at least one group generated significantly different AUC values ($F(2, 431) = 10.12$, $p < .001$). Post hoc comparisons using Tukey's HSD indicate that the EFT group demonstrated significantly higher ordinal AUC values than the HIT group (Mean difference = 0.11, 95% CI: 0.048 - 0.172, $p < .001$, Cohen's d = .52, 95% CI: 0.27 - 0.77) and the NCC group (mean difference = 0.091, 95% CI: 0.032 - 0.149, $p < .001$, Cohen's d = .44, 95% CI: 0.20 - 0.68). Additionally, no difference between the NCC and the HIT group was observed (mean difference = 0.018, 95% CI: -0.037 - 0.074, p = .7, Cohen's d = .09, 95% CI: -0.13 - 0.31).

Supplementary Figures

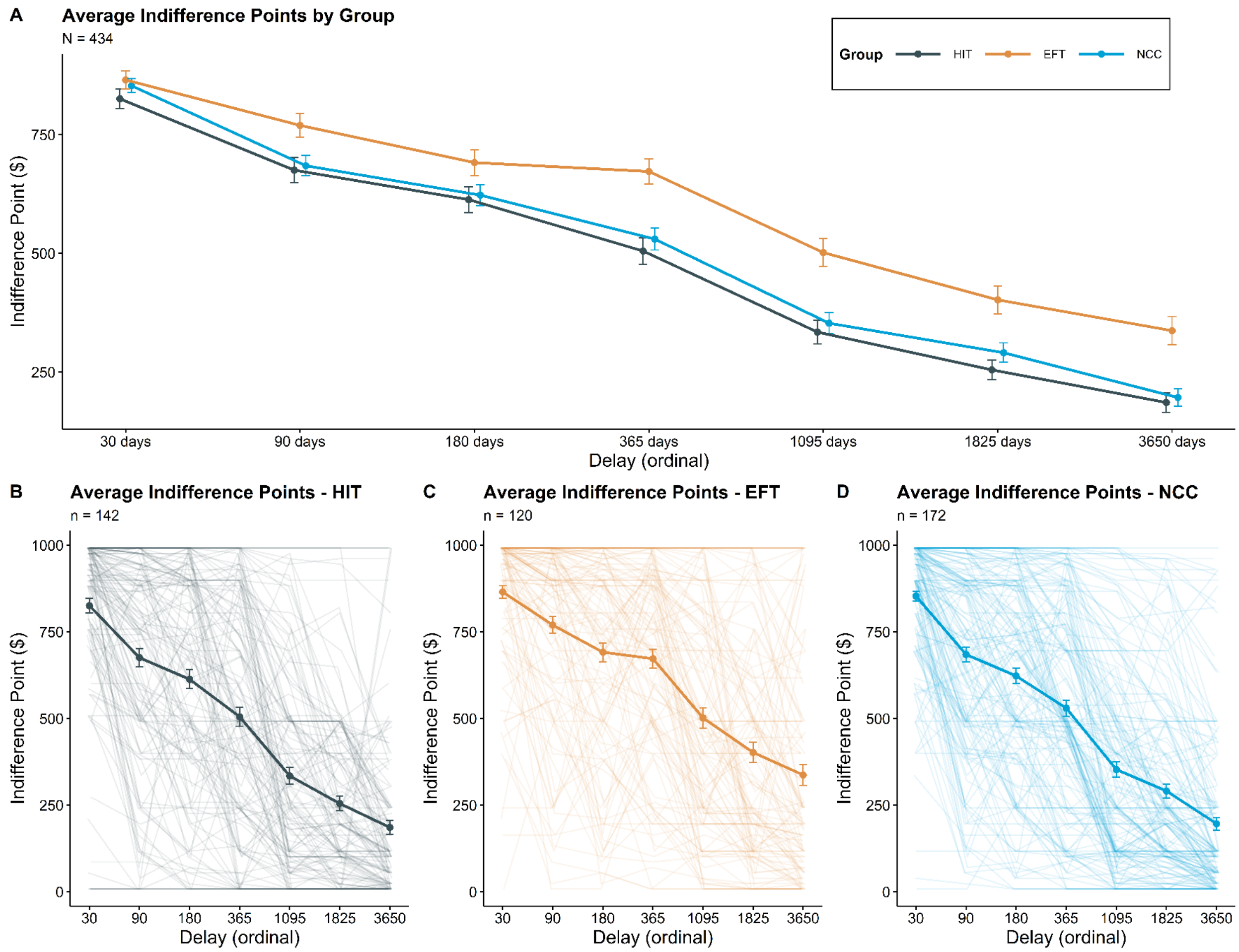

Supplementary Figure 1: Recreation of manuscript figure 2. Mean delay discounting curves of participants who completed the experiment. Panel A depicts mean indifference points as a function of delay by group assignment, plotted on an ordinal scale. Panels B, C, and D depict mean indifference points within each group; transparent lines depict individual subject indifference points. Error bars represent standard error.

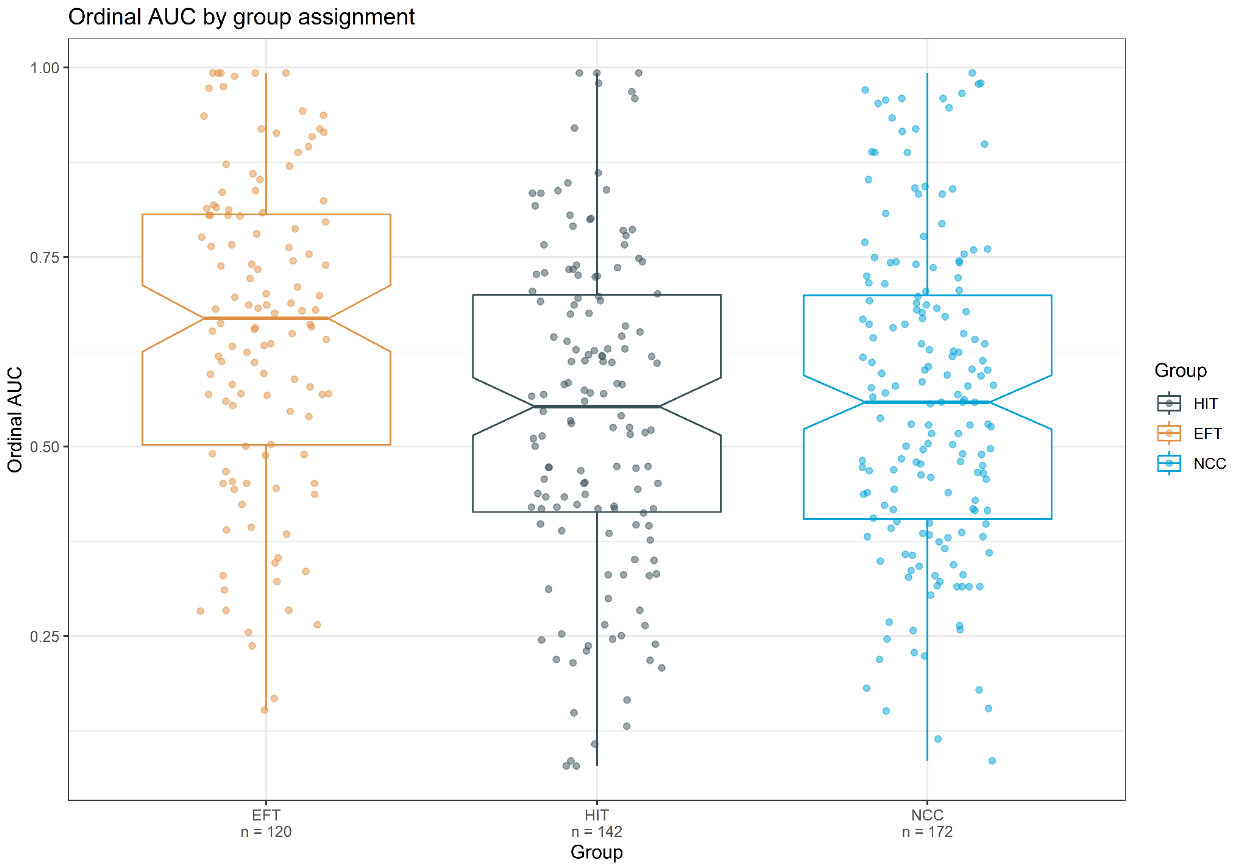

Supplementary Figure 2: Recreation of manuscript figure 3. Notched boxplot of grouped ordinal AUC values of participants who completed the experiment. Notches represent the median ± 1.58 * IQR/√n.
